# Aflatoxin B_1_ Conversion by Black Soldier Fly (*Hermetia illucens*) Larval Enzyme Extracts

**DOI:** 10.3390/toxins11090532

**Published:** 2019-09-12

**Authors:** Nathan Meijer, Geert Stoopen, H.J. van der Fels-Klerx, Joop J.A. van Loon, John Carney, Guido Bosch

**Affiliations:** 1 Wageningen Food Safety Research, Wageningen Campus P.O. Box 230, 6700 AE Wageningen, The Netherlands; geert.stoopen@wur.nl; 2 Wageningen University, Department of Plant Sciences, Laboratory of Entomology, Wageningen Campus P.O. Box 16, 6700 AA Wageningen, The Netherlands; joop.vanloon@wur.nl; 3 Mars, Incorporated, McLean, VA 22101, USA, john.carney@effem.com; 4 JMC Consulting, Portland, OR 972229, USA; 5 Wageningen University, Department of Animal Sciences, Animal Nutrition Group, Wageningen Campus P.O. Box 338, 6700 AH Wageningen, The Netherlands; guido.bosch@wur.nl

**Keywords:** aflatoxin, mycotoxin, black soldier fly, BSFL, *Hermetia illucens*, S9 fraction, cytochrome P450, metabolic conversion, enzyme induction

## Abstract

The larvae of the black soldier fly (*Hermetia illucens* L., BSFL) have received increased industrial interest as a novel protein source for food and feed. Previous research has found that insects, including BSFL, are capable of metabolically converting aflatoxin B_1_ (AFB_1_), but recovery of total AFB_1_ is less than 20% when accounting for its conversion to most known metabolites. The aim of this study was to examine the conversion of AFB_1_ by S9 extracts of BSFL reared on substrates with or without AFB_1_. Liver S9 of Aroclor-induced rats was used as a reference. To investigate whether cytochrome P450 enzymes are involved in the conversion of AFB_1_, the inhibitor piperonyl butoxide (PBO) was tested in a number of treatments. The results showed that approximately 60% of AFB_1_ was converted to aflatoxicol and aflatoxin P_1_. The remaining 40% of AFB_1_ was not converted. Cytochrome P450s were indeed responsible for metabolic conversion of AFB_1_ into AFP_1_, and a cytoplasmic reductase was most likely responsible for conversion of AFB_1_ into aflatoxicol.

## 1. Introduction

Aflatoxins are a group of mycotoxins that are primarily produced by the molds *Aspergillus flavus* and *Aspergillus parasiticus*. The four major aflatoxins are B_1_, B_2_, G_1_, and G_2_, which can be found in various food products such as peanuts and maize [1]. Aflatoxins are carcinogenic to humans (IARC Group 1) and a major economic and health problem globally, but especially in sub-Saharan Africa, Latin America, and Asia, since people and animals are exposed to levels that substantially elevate mortality and morbidity. Aflatoxin B_1_ (AFB_1_) has generated the most concern due to its toxicity and high contamination levels in food and feed commodities in certain areas such as Africa [2,3]. AFB_1_ is converted by animals and humans into a variety of metabolites, such as aflatoxin M_1_, Q_1_, P_1_, and aflatoxicol (AFL) [1,4]. AFB_1_ is a “procarcinogen” in the sense that hepatic microsomal cytochrome P450 (CYP450) enzymes convert AFB_1_ to AFB_1_-8,9-epoxide (AFBO), which has reactive and electrophilic properties that underlie the toxicity of the compound [5].

Although prevention of contamination of crops by aflatoxigenic molds is paramount, a variety of decontamination strategies have been developed. Postharvest detoxification methods for AFB_1_ include physical (heat and irradiation), chemical (acidification, ammoniation, and ozonation), and biological (whole organism or extracts thereof and enzymatic) treatments [6,7,8,9]. Although degradation levels of AFB_1_ are generally high for enzymatic treatments, treatment times are also high (up to several days), and there is uncertainty regarding the degradation products formed [6]. Since metabolites in treated products may still be toxic, determination of degradation products is a principal requirement for assessing the safety and efficacy of enzymatic detoxification treatments. Detoxification mechanisms are generally classified into three phases: “(I) introduction of reactive and polar groups into substrates through oxidation, reduction, or hydrolysis; (II) conjugation of metabolites with other compounds to create more polar or more easily excretable molecules; and (III) transport and elimination of compounds” [10]. Cytochrome P450 monooxygenase enzymes play a major role in the bioactivation of AFB_1_ in phase I metabolism [1,11]. These enzymes can be found in almost all (aerobic) organisms, but different P450 isoforms are species specific [12,13]. Some compounds may act as inhibitors of certain P450s. The best-known example of such an inhibitor is piperonyl butoxide (PBO) [13].

Insects have developed physiological and metabolic strategies to cope with potential toxic compounds, such as mycotoxins. Earlier work on the fruit fly *Drosophila melanogaster* Meigen (Diptera: Drosophilidae) [14,15,16,17] and on yellow mealworm (*Tenebrio molitor* L.; Coleoptera: Tenebrionidae; YMW) [18] has shown that some insects are capable of metabolizing AFB_1_. More recently, Bosch et al. (2017) [19] found that both black soldier fly larvae (*Hermetia illucens* L., BSFL) and YMW have a high AFB_1_ tolerance and that the toxin did not accumulate in these species. Moreover, the amount of AFB_1_ lost (from substrates to insects) varied from 83% to 95% for BSFL and 89% to 96% for YMW. However, the YMW formed AFM_1_ (present in the excreta) and AFB_1_ was detected in YMW when provided with feed containing 0.023 mg/kg of AFB_1_ or more. The concentration decreased when the YMW were starved before harvesting, which resulted in the larvae emptying their guts. This suggested that the gut contents contributed significantly to the measured AFB_1_ levels in the YMW. Camenzuli et al. (2018) [20] subsequently assessed the effects of a variety of mycotoxins, including AFB_1_, on BSFL and lesser mealworm (*Alphitobius diaperinus* Panzer; Coleoptera: Tenebrionidae). The mycotoxin metabolites AFL, aflatoxin P_1_, Q_1_, and M_1_ were taken into consideration in the chemical and bioaccumulation analyses. Mass balance calculations for BSFL suggested recovery of total AFB_1_ of less than 20%. Of the other analyzed metabolites, only AFL was detected at 0.2% of the overall mass balance; aflatoxin Q_1_, P_1_, and M_1_ were not detected (<0.001 mg/kg for larvae, <0.005 mg/kg for residual material (spiked feed and gut clean)).

In the corn earworm (*Helicoverpa zea* L.; Lepidoptera: Noctuidae), the toxicity of AFB_1_ depends on the CYP-mediated metabolic bioactivation [21]. Niu et al. (2008) [22] reported that dietary phytochemicals (i.e., xanthotoxin, coumarin, or indole-3-carbinol) induced midgut enzymes including CYP321A1 that can degrade AFB_1_ into mainly AFP_1_ and, to a lesser extent, an undefined metabolite. Feeding AFB_1_ without the phytochemical did not increase CYP321A1 transcripts and resulted in reduced growth and development, confirming that phytochemicals induced CYP enzymes that detoxify AFB_1_ [23]. Incubation of AFB_1_ with a homogenate of the larvae of the navel orangeworm (*Amyelois transitella* Walker; Lepidoptera: Pyralidae) resulted in the formation of mainly AFL and, to a lesser extent, aflatoxin B_2a_ and AFM_1_ [24]. This was in line with findings in testes of the fruit fly using a similar in vitro approach [17]. CYP6AB11 from navel orangeworm did not metabolize AFB_1_ [25]. Importantly, the in vitro study of Lee and Campbell (2000) [24] reported that PBO did not impact AFL formation by navel orangeworm, which suggested that this metabolite was formed by cytosolic NADPH-dependent reductase. Incubation of AFB_1_ with a homogenate of larvae of the codling moth (*Cydia pomonella* L.; Lepidoptera: Tortricidae) did not result in the metabolites AFL, AFB_2a_, and AFM_1_, which may relate either to absence of the metabolic system, different metabolic pathways, or that the system was not activated in the larvae, as these were not exposed to AFB_1_ before the study [24]. In honey bees (*Apis mellifera* L.; Hymenoptera: Apidae), there are also indications of P450-mediated metabolic detoxification of AFB_1_ [26].

In summary, BSFL have high tolerance to AFB_1_, and when AFB_1_ is provided in the feed, most of it cannot be recovered in the larvae and residual material. It is not clear whether and, if so, to what extent AFB_1_ is metabolically converted. As an alternative to live animals, an enzyme extract can be prepared to assess the potential for metabolic conversion of the species in vitro. In this manner, individual or several metabolic conversion pathways can be isolated and identified. The aim of this study was to examine the conversion of AFB_1_ by S9 extracts of BSFL reared on a substrate with AFB_1_. The S9 enzyme fraction contains both the membrane-bound as well as the soluble enzymes [27]. Liver S9 of Aroclor-induced rats was used as a reference. To investigate whether cytochrome P450 enzymes specifically are involved in the conversion of AFB_1_, PBO was tested in a number of treatments. We conclude that cytochrome P450s were indeed responsible for metabolic conversion of AFB_1_ into AFP_1_, and that a cytoplasmic reductase was most likely responsible for conversion of AFB_1_ into AFL.

## 2. Results

### 2.1. Effects of AFB_1_ in Feed on Larval Development

Live BSFL were subjected to two treatments, each applied in triplicate: one treatment in which the feed was spiked with AFB_1_ to a concentration of 0.5 mg/kg, and one control treatment without AFB_1_ added to the feed. Per replicate, 100 larvae less than 24 h old were provided with the feed and harvested after 9 days. Survival after these 9 days was high for both the control (average: 99.0) and the AFB_1_ treatment (average: 97.3) (*p* = 0.007). Average total biomass obtained was, respectively, 15.2 and 15.0 g (*p* = 0.685).

### 2.2. AFB_1_ Conversion by S9 Fractions

Table S1 shows the molar concentrations of AFB_1_ and the analyzed metabolites after incubation for all replicates. The results from the treatment with AFB_1_ but without S9 (−S9 + AFB_1_, t = 2 h) show that only AFB_1_ was found at the same concentration as what was spiked, and that no metabolites were formed. In the treatment with S9 but without AFB_1_ (+S9 − AFB_1_, t = 2 h), no AFB_1_ or metabolites were detected. This indicates that the AFB_1_ that was present in the larval feed was not converted into the analyzed metabolites by the larvae and did not accumulate.

**Figure 1:**
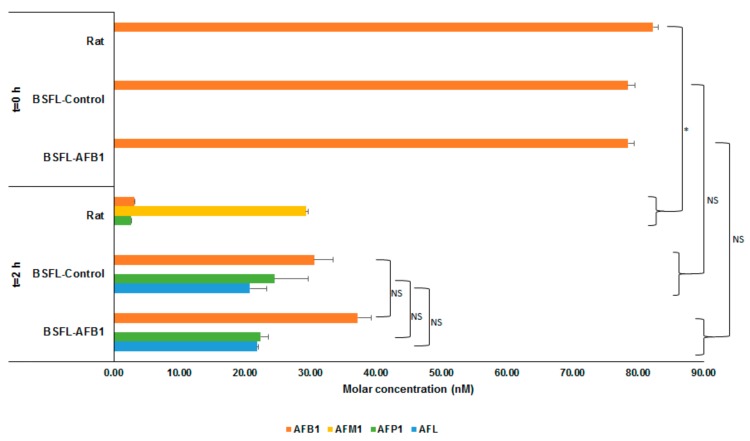
Molar concentrations (nM) of aflatoxin B_1_ (AFB_1_) and metabolites (AFM_1_, AFP_1_, and aflatoxicol (AFL)) for incubation of AFB_1_ with S9 fractions from rat liver, black soldier fly larvae (*Hermetia illucens* L., BSFL)-control, and BSFL-AFB_1_ after directly halting enzymatic activity (t = 0 h) and after 2 h of incubation. Significance of differences is indicated in the figure with * (*p* ≤ 0.05) or NS (not significant, *p* > 0.05).

Figure 1 shows the average molar concentrations (nM) of AFB_1_ and the analyzed metabolites for the three types of S9 fractions (rat, BSFL-control, and BSFL-AFB_1_) at two different points in time after addition of AFB_1_: after directly (t = 0 h) halting enzymatic activity (+S9 + AFB_1_, t = 0 h) and after incubation for 2 h (+S9 + AFB_1_, t = 2 h). For all three S9 fractions at t = 0 h, only AFB_1_ was present. The AFB_1_ concentration at t = 0 h was half of the concentration that was spiked at the start due to the addition of 100 μL of acetonitrile to the 100 μL mixture of Regensys A buffer, NADPH, AFB_1_, and S9. The results of the BSFL-control and BSFL-AFB_1_ S9 fractions that were incubated for 2 h show that part of the AFB_1_ was converted into AFP_1_ (23.44 nM, *p* = 0.847) and AFL (21.32 nM, *p* = 0.824). The total molar concentrations (AFB_1_ + AFP_1_ + AFL) of these two treatments were equal to the total molar concentration of AFB_1_ in the t = 0 h treatments (BSFL-control: *p* = 0.275; BSFL-AFB_1_: *p* = 0.211). This indicates that no metabolic conversion occurred other than the type that was observed (i.e., AFB_1_ into AFP_1_ and AFL).

The results of the rat S9 treatments that were incubated for 2 h show that AFM_1_ (29.34 nM) and, to a lesser extent, AFP_1_ (2.59 nM) had formed. The amount of AFB_1_ that was recovered after incubation from the treatment with the rat S9 fraction (3.15 nM) was less than what was recovered from the BSFL treatments (BSFL-AFB_1_: 37.23 nM; BSFL-control: 30.57 nM). In addition, the total molar concentration of AFB_1_ and analyzed metabolites for the rat S9 treatment after incubation for 2 h (35.17 nM) was less than the total AFB_1_ molar concentration for the rat S9 treatment at t = 0 h (82.29 nM). This indicates that some of the spiked AFB_1_ was converted by the rat S9 into different metabolites than those that have been analyzed.

### 2.3. Effect of PBO on AFB_1_ Conversion by S9 Fractions

**Figure 2:**
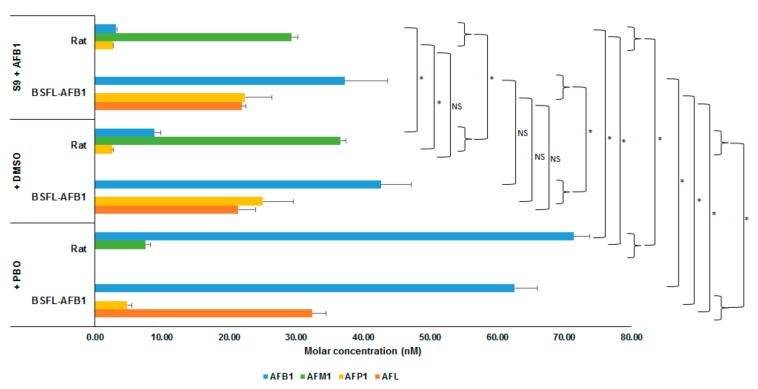
Molar concentrations (nM) of AFB_1_ and metabolites (AFM_1_, AFP_1_, and AFL) for incubation of AFB_1_ with S9 fractions from rat liver and BSFL-AFB_1_, and with dimethyl sulfoxide (DMSO) or DMSO + cytochrome P450 inhibitor piperonyl butoxide (PBO) added, after 2 h of incubation. Significance of differences is indicated in the figure with * (*p* ≤ 0.05) or NS (not significant, *p* > 0.05).

Figure 2 shows the average molar concentrations (nM) of AFB_1_ and the analyzed metabolites for the two types of S9 fractions (rat and BSFL-AFB_1_) after incubation with AFB_1_ for 2 h. One treatment contained an S9 fraction (rat or BSFL-AFB_1_) and AFB_1_ (+S9 + AFB_1_, t = 2 h); the second also contained dimethyl sulfoxide (DMSO, (+S9 + AFB_1_ + DMSO, t = 2 h); and in the third, PBO (dissolved in DMSO) was added to the S9 fractions and AFB_1_ (+S9 + AFB_1_ + DMSO + PBO, t = 2h). For the AFB_1_ larvae S9 treatments, the differences between the treatment containing DMSO and the treatment without further additives were not significant for each included metabolite (AFB_1_ (*p* = 0.296), AFL (*p* = 0.758), AFP_1_ (*p* = 0.491)). This indicates that the DMSO in which the PBO was dissolved did not affect the conversion of the BSFL-AFB_1_ S9 fraction. Compared with the BSFL treatment without additional additives, the AFP_1_ concentration in the PBO treatment was reduced (*p* = 0.002), while the AFL (*p* = 0.001) and AFB_1_ (*p* = 0.004) concentrations were elevated. Comparing the rat treatment with PBO to the treatment without additives shows that the conversion into AFP_1_ was completely halted and the conversion into AFM_1_ was reduced (7.54 nM). The AFB_1_ molar concentration was higher in the PBO treatment than in the treatment without additives, but the total molar concentration of the analyzed metabolites in the PBO treatment was equal to the total AFB_1_ molar concentration at t = 0 h (+S9 + AFB_1_, t = 0 h; *p* = 0.129). This indicates that the PBO halted the conversion of AFB_1_ by rat S9 into different metabolites than those that have been analyzed.

## 3. Discussion and Conclusions

Body weight and survival of control larvae and larvae exposed to AFB_1_ were similar. We therefore conclude that the BSFL were unaffected by the addition of AFB_1_ to their feed, which is in line with the findings of Bosch et al. (2017) [19] and Camenzuli et al. (2018) [20].

The study showed that S9 preparations of BSFL converted approximately 60% of the AFB_1_ to AFL and AFP_1_. The remaining 40% of AFB_1_ was not converted into the analyzed metabolites. The amounts of AFL and AFP_1_ were more or less equal, and there was no difference in activity of S9 prepared from larvae grown on substrates with or without AFB_1_. This suggests that the enzymes involved in the biotransformation of AFB_1_ are part of constitutive detoxification systems of the BSFL. Activation of the system in the larvae via pre-exposure—as hypothesized by Lee and Campbell (2000) [24], discussed above—is therefore not required for the system’s functioning.

The addition of cytochrome P450 inhibitor PBO partially inhibited the formation of AFP_1_ by BSFL S9 extracts, indicating that a P450 enzyme is involved in the conversion from AFB_1_ into AFP_1_. Conversion to AFL by the BSFL S9 fraction was not inhibited when PBO was added, indicating that it is not catalyzed by P450 enzymes. The total recovery of AFB_1_ and metabolites in the BSFL PBO treatment exceeded the total molar concentration of metabolites in the treatment without additives at approximately 122% (*p* = 0.001), but this was within the range of 2 * SD.

**Figure 3:**
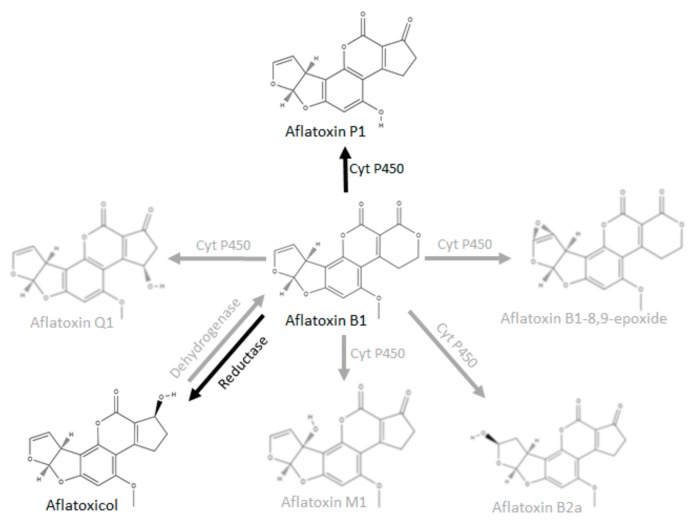
Selected metabolic conversion pathways known for AFB_1_ (adapted from Lee and Campbell, 2000 [24] and Dohnal et al., 2014 [5]).

Since AFB_1_ is converted to AFL by a cytosolic NADPH-dependent reductase [24,28,29], we therefore propose that this conversion to AFL by BSFL occurs via the same pathway. Figure 3 shows selected metabolic conversion pathways known for AFB_1_. The black arrows denote metabolic pathways that have been found to be active in BSFL S9 fractions in this study; the grey arrows denote known pathways in other species.

The reaction from AFB_1_ to AFL is reversible. The cofactor for the reduction of AFB_1_ is NADPH, which was added to the AFB_1_ at the start of the trials, together with the Regensys A regenerating system. The cofactor for the dehydrogenation of AFL yielding AFB_1_ is NADP, which accumulates when the regeneration of NADPH stops [28]. It cannot be ruled out that BSFL possess this microsomal dehydrogenase, which would revert the reaction and increase the level of AFB_1_ again, thereby negating detoxification. This reversion could, for instance, occur in case of an incubation time longer than 2 h or in the absence of an NADPH regenerating system. AFM_1_ was not formed by the BSFL S9 fraction in this study. The latter conversion is catalyzed by the cytochrome P450 enzyme CYP1A2 [5]. The absence of the formation of AFM_1_ in the BSFL treatment (in this study as well as in Camenzuli et al., 2018 [20]) and its presence in the treatment with rat S9 suggests the absence of this enzyme in BSFL. However, the enzyme may also have been deactivated during preparation of the BSFL S9 fraction.

Compared with the conversion of AFB_1_ by live BSFL, as studied by Camenzuli et al. (2018) [20], there are a few major differences in how the AFB_1_ was metabolized by the S9 fraction observed in this study. Firstly, no aflatoxin P_1_, Q_1_, and M_1_ could be recovered by Camenzuli et al. (2018) [20], and the amount of AFL was negligible (0.2% of mass balance). In the current study, however, approximately equal proportions of AFL and AFP_1_ were recovered. Moreover, while less than 20% of AFB_1_ could be recovered in the mass balance of Camenzuli et al. (2018) [20], 100% could be recovered in this study. It is unclear what the exact reasons are for these discrepancies, but the following hypotheses may be considered. Since live larval cells are expected to contain a wider variety of cofactors (other than NADPH, as used in conjunction with the S9 fraction in this study), a larger number of enzymes may be activated. It is possible that enzymes in live larvae first convert the AFB_1_ into AFL and AFP_1_, which, in turn, are precursors for other compounds. These may, for instance, be reactive metabolites that bind to other proteins. A second option is that the conversion of AFB_1_ into AFL and AFP_1_ in the S9 fraction is accelerated due to the absence of other cofactors that would catalyze different metabolic pathways. More research on the exact pathways of AFB_1_ conversion by live BSFL is recommended in order to identify and quantify degradation products so that the efficacy and safety of reared larvae can be assessed. This could, for instance, be achieved by performing the analyses described in this manuscript with inhibitors of specific cytochrome P450 and/or NADPH-dependent reductase enzymes.

In conclusion, BSFL S9 fractions converted AFB_1_ into AFP_1_ and AFL. Furthermore, exposing BSFL to AFB_1_ did not impact the conversion capacity, suggesting that the enzymes involved are part of a general metabolic system. No other analyzed metabolites were formed. Cytochrome P450s were responsible for metabolic conversion of AFB_1_ into AFP_1_. A cytoplasmic reductase was most likely responsible for conversion of AFB_1_ into AFL.

## 4. Materials and Methods

**Figure 4:**
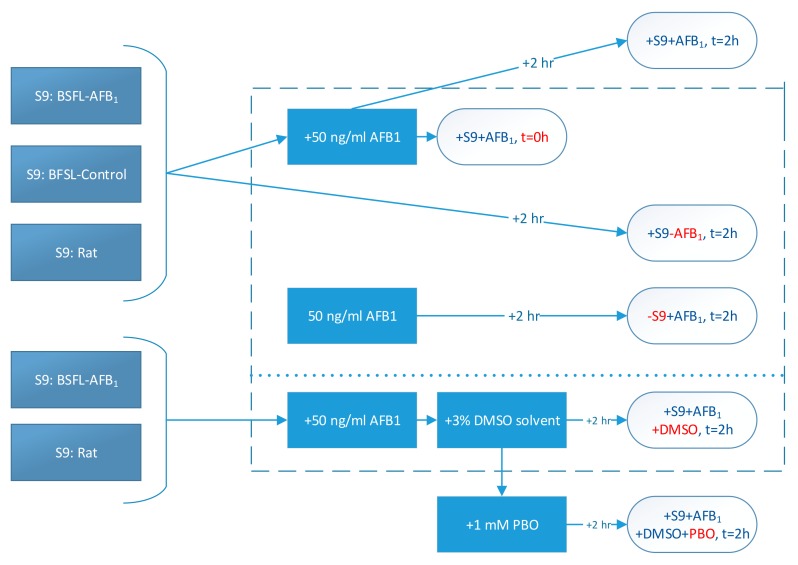
Schematic representation of treatments (control treatments within dotted lines; red letters indicate the difference between that treatment and the + S9 + AFB_1_, t = 2 h treatment).

The overall methodology for the different treatments is shown schematically in Figure 4. Firstly, BSFL were reared on feed that had been spiked with AFB_1_ (0.5 mg/kg). A second batch of larvae was reared on noncontaminated feed as a control. From these two batches of larvae, separate S9 fractions were prepared, and a commercial rat S9 fraction was used for comparison. These S9 fractions were incubated with AFB_1_ for 2 h (+S9 + AFB_1_, t = 2 h). In addition, an incubation was included in which PBO dissolved in DMSO was added to the mixture of the S9 fraction and AFB_1_ (+S9 + AFB_1_ + DMSO + PBO, t = 2 h). Four control treatments were used in this study. Firstly, acetonitrile was directly added to an S9 and AFB_1_ mixture at t = 0 h in order to halt enzymatic activity (+S9 + AFB_1_, t = 0 h). Secondly, one mixture was prepared excluding S9 fractions (−S9 + AFB_1_, t = 2 h), and a third control treatment excluded AFB_1_ (+S9 − AFB_1_, t = 2 h). Finally, a solvent control treatment containing DMSO was used (+S9 + AFB_1_ + DMSO, t = 2 h).

Differences between treatments were tested for significance by multiple one-way ANOVA tests (α = 0.05) using the Analysis ToolPak add-in for Microsoft Excel 2016 MSO 32-bit (version 16.0.4849.1000, Microsoft Corporation, Redmond, WA, United States of America, 2016). This was done by comparing the molar concentrations of individual (AFB_1_, AFM_1_, AFP_1_, and AFL) and total metabolites between treatments.

### 4.1. Larvae Treatment

A standard dry wheat-based mash feed (layer meal based) was spiked with AFB_1_ (*A. flavus*, 99.6% purity, Sigma-Aldrich, Saint Louis, MO, USA) by an external laboratory (Ducares B.V., Utrecht, The Netherlands) to reach a concentration of 0.5 mg/kg of feed. This was the highest concentration used by Bosch et al. (2017) [19], which had no effect on the mortality and growth of the larvae. The feed used was the same batch that was used by Camenzuli et al. (2018) [20], which had been prepared by Ducares B.V., Utrecht, The Netherlands. A sample of the nonspiked feed was used as a control.

Per dietary treatment, three plastic boxes (17.8 × 11.4 × 6.5 cm) were prepared for each replicate. Each box contained 18 g (±0.1 g) of feed, which was manually mixed with 25 mL (±0.1 mL) of tap water. One hundred larvae less than 24 h old and originating from the BSF colony maintained at the Laboratory of Entomology (Wageningen University, Wageningen, The Netherlands) were added to the box. The box was then closed with a perforated lid. All boxes were kept in a climate cabinet (27 ± 1 °C and 88% ± 1% relative humidity) for 9 days. After 9 days, the larvae were collected and counted. The larvae were cleaned by rinsing with lukewarm tap water, dried with paper, and snap-frozen in liquid nitrogen. Larvae were stored at −80 °C until further analyses.

### 4.2. Preparation of S9 Fraction

Frozen larvae were ground to a fine powder with a precooled mortar and pestle under addition of liquid nitrogen. The frozen powder was transferred to a precooled polypropylene tube (50 mL Greiner, VWR, Amsterdam, The Netherlands) and 1.5 mL of ice cold buffer (1.15% KCl in 50 mM Tris/HCl pH 7.4) was added per gram of powder (7 g of powder + 10.5 mL of buffer). The sample was mixed thoroughly by tapping on the bench to bring the powder in contact with the buffer. Care was taken that the powder did not thaw before it was mixed with the extraction buffer. After obtaining a homogenous suspension, the material was further extracted by gently inverting the tubes 100 times. The suspensions were centrifuged in a precooled rotor for 25 min at 8960 rcf and 4 °C. The supernatants were collected, pooled, and mixed. Then, 500 μL aliquots were snap-frozen in liquid nitrogen and stored at −80 °C.

The protein concentration was determined using the DC Protein assay (Bio-Rad Laboratories, Veenendaal, The Netherlands) according to the manufacturer’s protocol and bovine serum albumin (BSA) was used as a standard. The protein content of the insect S9 fractions was on average 36 mg/mL. The protein content of the rat liver S9 was 38 mg/mL (data provided by the manufacturer).

### 4.3. S9 Incubations with AFB_1_

Samples were prepared on ice and contained 1× Regensys A buffer (Moltox, Boone, USA), 5 mM NADPH, 50 ng/mL AFB_1_ (50 μg/kg), and 2.5 mg/mL S9 protein in a final volume of 100 μL. NADPH was prepared freshly in Regensys A buffer. AFB_1_ was dissolved in DMSO and dilutions in Regensys buffer were prepared prior to the incubations. The final concentration of DMSO in the assay was 0.03%. The reactions were started by addition of S9 to the mixture and transferring the tubes to 37 °C in an Eppendorf thermomixer. Most samples were incubated for 2 h. t = 0 samples were prepared by adding 100 μL of cold acetonitrile prior to addition of S9.

To study the role of cytochrome P450 enzymes in the conversion of AFB_1_, 1 mM of PBO (or 3% DMSO as solvent control) was included in the S9 mixes. Samples were incubated for 2 h and the reactions were stopped by addition of 100 μL of ice cold acetonitrile. Samples were vortexed thoroughly, put on ice for 5 min, and finally stored at −80 °C.

### 4.4. Chemicals

Regensys A buffer and rat liver S9 (Aroclor-induced rats; lyophilized S9 preparation) were purchased from Trinova Biochem (Gießen, Germany). Regensys A buffer consists of 100 mM phosphate buffer pH 7.4, 33 mM KCl, 8 mM MgCl_2_, and 5 mM glucose-6-phosphate (NADPH regeneration system). NADPH, AFB_1_, AFM_1_, DMSO-HybriMax, and PBO were purchased from Sigma-Aldrich (Zwijndrecht, The Netherlands); AFP_1_ from TRC (Toronto Research Chemicals, North York, Canada); and AFL from Enzo Life Sciences BVBA, (Brussels, Belgium). Potassium chloride was obtained from Merck (Amsterdam, The Netherlands) and Tris-buffer from Fisher Scientific (Landsmeer, The Netherlands).

### 4.5. LCMS Analyses

Analyses were performed in largely the same way as in Camenzuli et al. (2018) [20]. Samples were defrosted, vortexed, and centrifuged for 5 min, 14,000 rpm at room temperature. From the supernatant, 190 μL was transferred to an LCMS vial and 10 μL of ^13^C-labeled internal standard solution was added. Samples were mixed and 5 μL was analyzed with an LC-MS/MS-based method for the analysis of mycotoxins in feed and food materials. The accredited scope of this method was extended in order to also quantify the AFB_1_ and its metabolites in larvae and residual material (excreta and residual feed) of BSFL.

Two MRM transitions were included for each metabolite in the MS/MS method. Details on this and additional MS/MS settings can be found in Tables S2 and S3 of the Supplementary Materials. Each metabolite was identified by its retention time and the peak area ratio between two transitions: the quantifier and the qualifier. Quantification was performed by bracketed calibration (an interval of not more than 10 injections) on the peak area of the quantifier (qn) of calibration solutions in solvent. Concentrations of AFB_1_ and metabolites were corrected for matrix effects with the use of their respective ^13^C-isotope-labeled standards (AFB_1_ and AFM_1_) or by means of matrix-matched calibration standards (AFP_1_ and AFL).

The limit of quantification (LOQ) was defined as the lowest calibrated level which complied with the required QC parameters as mentioned in SANTE/11945/2015. Metabolite-specific LOQs can be found in Table S4 in the Supplementary Materials.

The LC-MS/MS system consisted of an injection and pump system from Waters (Waters, Milford, MA) and an AB Sciex QTRAP 6500 triple quad system equipped with an electrospray ionization (ESI) source operated in positive mode (AB Sciex, Nieuwerkerk a/d IJssel, The Netherlands). For LC separation, a 100 × 2.1 mm ID, 3 µm Restek Ultra Aqueous C18 column (Interscience, Breda, The Netherlands) was used. Details on the LC-MS/MS settings can be found in Table S5 of the Supplementary Materials. The LC eluent gradients were 1 min isocratic at 100% A, followed by a linear gradient to 100% B in 4 min. For complete elution of all matrix coextractants from the column, the final composition at 100% B was kept for 2 min. In 30 s, the initial conditions were restored and then equilibrated for 2 min prior to the next injection.

## Supplementary Materials

The following are available online at https://www.mdpi.com/2072-6651/11/9/532/s1. Table S1. Molar concentrations (nmol/L) of aflatoxin B_1_ (AFB_1_) and analyzed metabolites (aflatoxicol (AFL), aflatoxin P_1_ (AFP_1_), and aflatoxin M_1_ (AFM_1_)) after incubation. Results of individual replicates. Table S2. MS/MS parameters. Table S3. MS/MS transitions. Table S4. LOQs of analyzed compounds. Table S5. LC-MS/MS parameters.
