# Supplementary material for: Aflatoxin B1 Conversion by Black Soldier Fly (Hermetia illucens) Larval Enzyme Extracts
Source: Toxins (Basel). 2019 Sep 12;11(9):532. doi: 10.3390/toxins11090532 (PMC6784232; doi:10.3390/toxins11090532)
Supplement: Supplementary file 1 [file toxins-11-00532-s001.pdf]

Supplementary Materials: Aflatoxin B_1 Conversion by Black Soldier Fly (*Hermetia illucens*) Larval Enzyme Extracts

Nathan Meijer, Geert Stoopen, H.J. van der Fels-Klerx, Joop J.A. van Loon, John Carney and Guido Bosch

Table 1. Molar concentrations (nmol/L) of aflatoxin B_1 (AFB_1) and analyzed metabolites (aflatoxicol (AFL), aflatoxin P_1 (AFP_1), and aflatoxin M_1 (AFM_1)) after incubation. Results of individual replicates.

Treatment	S9 Fraction	Replicate Number	Concentration (nmol/L)			
			AFB_1	AFL	AFP_1	AFM_1
+S9 + AFB_1, t = 2h	BSFL-control	1	40.26	28.02	7.91	-
		2	29.69	22.05	23.30	-
		3	21.77	12.26	42.26	-
	BSFL-AFB_1	1	43.45	21.22	18.73	-
		2	37.56	21.98	21.75	-
		3	30.66	22.42	26.68	-
	Rat	1	2.97	-	2.61	28.74
		2	3.26	-	2.74	28.94
		3	3.20	-	2.71	30.33
+S9 + AFB1, t = 0 h	BSFL-control	1	82.34	-	-	-
		2	76.31	-	-	-
		3	76.52	-	-	-
	BSFL-AFB_1	1	79.64	-	-	-
		2	80.71	-	-	-
		3	75.05	-	-	-
	Rat	1	79.43	-	-	-
		2	83.60	-	-	-
		3	83.84	-	-	-
+S9 − AFB_1, t = 2 h	BSFL-control	1	-	-	-	-
		2	-	-	-	-
		3	-	-	-	-
	BSFL-AFB_1	1	-	-	-	-
		2	-	-	-	-
		3	-	-	-	-
	Rat	1	-	-	-	-
		2	-	-	-	-
		3	-	-	-	-
−S9 + AFB_1, t = 2 h	N/A	1	152.71	-	-	-
		2	163.06	-	-	-
		3	157.78	-	-	-
+S9 + AFB_1 + DMSO, t = 2 h	BSFL-AFB_1	1	65.30	34.13	4.13	
		2	63.63	30.26	4.88	
		3	58.80	32.90	5.42	
	Rat	1	68.79	-	-	8.16

		2	73.68	-	-	6.84
		3	71.63	-	-	7.63
**+S9 + AFB$_1$ + DMSO + PBO, t = 2 h**	**BSFL-AFB$_1$**	1	44.99	24.15	21.18	-
		2	45.47	18.89	23.90	-
		3	37.47	21.03	30.06	-
	**Rat**	1	9.88	-	2.60	37.60
		2	8.25	-	2.74	36.31
		3	8.48	-	2.30	36.02

- denotes that the concentration was below the limit of quantification (LOQ).

Table 2. MS/MS parameters.

Parameter	Settings
Scan type	MRM
Scheduled MRM	No
Dwell time	5
Resolution Q1	unit
Resolution Q3	unit
Settling time	5 ms
MR pause	5 ms
Curtain gas (CUR)	40
Collision gas (CAD)	Medium
IonSpray voltage (IS)	5000 V
Temperature (TEM)	400 °C
Ion source gas 1 (GS1)	50
Ion source gas 2 (GS2)	50

Table 3. MS/MS transitions.

Component	Q1 (*m*/*z*)	Q3 (*m*/*z*)	DP (V)	EP (V)	CE (V)	CXP (V)
Aflatoxin B1 (ql)	313.0	128.1	40	10	91	10
Aflatoxin B1 (qn)	313.0	285.2	40	10	33	16
Aflatoxicol (qn)	297.1	269.0	66	10	29	16
Aflatoxicol (ql)	297.1	141.0	66	10	65	14
Aflatoxin M1 (qn)	328.9	272.9	61	10	33	18
Aflatoxin M1 (ql)	328.9	229.0	61	10	55	16
Aflatoxin P1 (qn)	299.0	271.0	101	10	33	18
Aflatoxin P1 (ql)	299.0	114.9	101	10	71	12
Aflatoxin Q1 (qn)	329.1	310.8	71	10	29	16
Aflatoxin Q1 (ql)	329.1	177.0	71	10	45	22
13C17-Aflatoxin B1	330.1	301.1	40	10	33	16
13C17-Aflatoxin M1	346.1	288.1	61	10	33	18
Aflatoxin B2 (qn)	315.1	287.2	40	10	37	18
Aflatoxin B2 (ql)	315.1	259.2	40	10	43	18
13C17-Aflatoxin B2	332.1	303.1	40	10	37	18
Aflatoxin G1 (qn)	329.0	243.2	40	10	39	14
Aflatoxin G1 (ql)	329.0	200.0	40	10	53	12
13C17-Aflatoxin G1	346.1	257.1	40	10	39	14
Aflatoxin G2 (qn)	331.1	313.2	40	10	35	18

Aflatoxin G2 (ql)	331.1	245.2	40	10	43	14
13C17-Aflatoxin G2	348.1	259.1	40	10	43	14

Table 4. LOQs of analyzed compounds.

Compound	LOQ (ng/mL)
Aflatoxin B1	0.25
Aflatoxin B2	10
Aflatoxin P1	5
Aflatoxin Q1	5
Aflatoxin M1	0.125
Aflatoxicol	1.25
Aflatoxin G1	0.125
Aflatoxin G2	0.125

Table 5. LC-MS/MS parameters.

Parameter	Settings
Sample temperature	10 °C
Temperature column	35 °C
Injection volume	5 µL
Mobile phase (A)	1 mM ammonium formate and 1% formic acid in water
Mobile phase (B)	1 mM ammonium formate and 1% formic acid in methanol:water—95:5
LC flow rate	400 µL/min
Electrospray ionization (ESI) temperature	400 °C
ESI spray voltage	5.0 kV
Ion source gas 1 (GS1)	50
Ion source gas 2 (GS2)	50
